# Comparative Study on Dynamic Compression Behaviors of Steel Fiber-Reinforced Cementitious Composites and Steel Fiber-Reinforced Concrete at Elevated Temperatures

**DOI:** 10.3390/ma19020238

**Published:** 2026-01-07

**Authors:** Fengzeng Li, Zichen Wang, Liang Li, Bo Zhao

**Affiliations:** 1 School of Civil Engineering and Architecture, Beijing Jiaotong University, Beijing 100044, China; 2 Key Laboratory of Urban Security and Disaster Engineering, Beijing University of Technology, Ministry of Education, Beijing 100124, China

**Keywords:** steel fiber-reinforced cementitious composites (SFRCC), steel fiber-reinforced concrete (SFRC), dynamic compression behaviors, high temperatures, split Hopkinson pressure bar (SHPB)

## Abstract

**Highlights:**

**What are the main findings?**

- SFRCC maintains progressive failure and ~40% strength at 800 °C; SFRC deteriorates rapidly post-600 °C.
- SFRCC specimens with a 2% fiber content exhibited stable strain-rate sensitivity and a fiber pull-out-dominated failure mode when subjected to continuously increasing loads at 600 °C.
- SFRCC excels in stress/toughness at 200–400 °C; SFRC outperforms in 600–800 °C due to aggregate stability.

**What are the implications of the main findings?**

- Supports using SFRCC in structures requiring high-temperature ductility and fire resistance.
- Highlights importance of fiber–matrix interface stability for dynamic performance in fire conditions.
- Guides material selection: SFRCC for low–medium and SFRC for medium–high temperature applications.

**Abstract:**

This study presents a comparative investigation of the dynamic compression behaviors of steel fiber-reinforced cementitious composites (SFRCC) and steel fiber-reinforced concrete (SFRC) under elevated temperatures up to 800 °C, utilizing a split Hopkinson pressure bar (SHPB). The experimental results demonstrate that SFRCC exhibits enhanced overall performance at high temperatures, maintaining a progressive failure mode and approximately 40% residual strength even at 800 °C, while SFRC experiences rapid deterioration beyond 600 °C. In the low-to-medium temperature range of 200–400 °C, SFRCC shows significantly higher dynamic peak stress and toughness compared to SFRC. However, in the high-temperature range of 600–800 °C, the superior thermal stability of the aggregate–matrix system in SFRC results in better performance in these metrics. The findings provide insights into the damage evolution mechanisms of fiber-reinforced cement-based materials under coupled thermal and dynamic loads, offering a critical theoretical foundation for material selection in engineering structures exposed to extreme thermal environments.

## 1. Introduction

Engineered cementitious composites (ECC) possess a distinctive microstructure and material design that enable them to achieve a tensile strain limit of 3–8%, which is significantly higher than the 0.01% limit of conventional concrete [1]. ECC exhibits outstanding crack control capabilities, effectively maintaining crack widths within 100 μm [2,3]. Global warming has led to more frequent high-temperature weather. This situation is further exacerbated by the growing prevalence of high-rise buildings and underground spaces during urbanization. Together, these factors amplify the risks associated with fires. Traditional building materials, when subjected to extreme conditions like fires and explosions, are prone to cracking due to thermal stresses, posing a significant threat to structural safety [4] (pp. 330–344). Incorporating reinforcing fibers into ECC and concrete is a crucial approach to enhancing their resistance to cracking and improving fire performance [5]. ECC utilizes high-strength fibers to bridge cracks. This mechanism allows the material to undergo multiple cracking events while avoiding brittle failure [6]. Among the various fiber-reinforced materials, steel fibers stand out due to their higher elastic modulus, significantly improving the tensile strength and impact resistance of the matrix [7]. Additionally, their exceptional high-temperature resistance allows them to effectively bridge cracks and maintain residual strength in fire conditions [8]. Steel fibers have thus become the ideal choice for enhancing the thermal resistance of ECC and concrete materials, holding an irreplaceable position in civil engineering applications where both mechanical performance and economic benefits are critical [9,10]. Steel fiber-reinforced cementitious composites (SFRCC) and steel fiber-reinforced concrete (SFRC) are widely utilized in industrial buildings, bridges, tunnels, and other engineering projects [11,12,13]. Research by Caverzan et al. [14] (pp. 339–346) shows that adding steel fibers increases the static peak strength of ECC by approximately 40%. Under elevated strain rates, the crack-arresting influence of steel fibers may be reduced. Studies by Ahmed et al. [15] (pp. 1088–1097) and Li et al. [16] reveal that steel fibers significantly reduce the static brittle nature of ECC materials, and under dynamic loading, they demonstrate excellent strength enhancement and energy absorption capabilities.

As fire resistance requirements for modern building structures continue to increase, extensive studies on the high-temperature behavior of various ECCs have been conducted both domestically and internationally [17] (pp. 1–31). Experimental investigations by Bhat et al. [18] (pp. 370–380) and Sahmaran et al. [19] (pp. 297–304) show that after one hour of exposure to temperatures of 200 °C, 400 °C, and 600 °C, ECC loses moisture at rates of 7.34%, 12.68%, and 13.35%, respectively. Under these conditions, its compressive strength decreases to 85.8%, 85.4%, and 53.2% of the value at room temperature. The constituent materials in ECC have different thermal expansion coefficients. Under high-temperature exposure, this difference causes microcracks to develop within the aggregates. These microcracks lead to internal stresses, resulting in a further reduction in the overall strength [20].

Compared with polymer fibers such as PVA and PE, which are prone to severe performance degradation at high temperatures [21,22,23], the steel fibers used in this study, owing to their excellent thermal resistance, represent an effective option for enhancing the performance of ECC under extreme temperature conditions. However, when steel fibers are exposed to temperatures above 300 °C, an oxide layer forms on their surface, weakening the bond strength between the fibers and the cement matrix [24] (pp. 1106–1118). Under high-temperature conditions, the hydration products within ECC may decompose [25]. Furthermore, the interaction mechanisms between the resulting loosely structured microstructure and the steel fibers remain a significant research gap. Additionally, most current high-temperature experimental studies use quasi-static loading methods, which fail to accurately simulate dynamic loading conditions, such as impact or explosions, that could occur in real engineering applications. As a result, there is a limited understanding of the damage evolution mechanisms of SFRCC under high-temperature dynamic coupling. In conclusion, while some insights into the high-temperature performance of SFRCC and SFRC have been gained, there is a scarcity of systematic comparative studies on their high-temperature dynamic behavior under identical fiber contents. Specifically, the variations in strain-rate sensitivity, energy absorption mechanisms, and the critical temperature dictating the shift from ductile to brittle failure remain incompletely understood. Through systematic comparative experiments, this paper aims to elucidate the differences and underlying mechanisms between SFRCC and SFRC under high-temperature dynamic compression, addressing the existing research gap. The findings provide a direct theoretical basis and data support for material selection in engineering structures exposed to high-temperature environments.

This paper adopts a research framework comprising experiments, analysis, and conclusions to systematically investigate the high-temperature dynamic mechanical properties of SFRCC and SFRC. Section 2 provides a comprehensive description of the mix design, preparation process, heating regimen, and dynamic compression testing procedure for both SFRCC and SFRC specimens. Section 3, based on experimental data, focuses on comparing and analyzing the dynamic strength degradation patterns, energy absorption characteristics, and differences in the failure modes of the two materials under high-temperature conditions, highlighting the impact of strain rate, fiber content, and matrix properties on high-temperature performance. Section 4 offers a systematic summary of experimental phenomena and quantitative analysis results, identifying key influencing factors and optimization strategies for improving the high-temperature dynamic mechanical properties of SFRCC and SFRC.

## 2. Materials and Methods

### 2.1. Specimen Preparation

**Figure 1:**
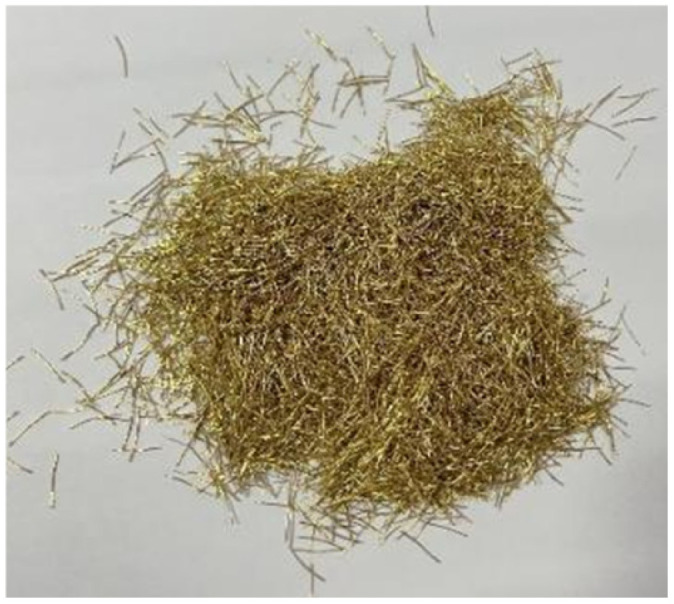
Copper-coated steel fiber.

The raw materials used for the SFRCC specimens include P.O. 42.5 ordinary Portland cement, silica fume, Class II medium sand (particle size distribution: 0.01–4 mm, fineness modulus: 2.7), water, polycarboxylate high-performance water reducer, and copper-plated steel fibers (Figure 1). The physical and mechanical properties of the fibers are presented in Table 1. Following multiple trial mixes and referencing previous studies [26], the optimal simple mass ratio for the SFRCC specimens was determined, as shown in Table 2. In the specimen numbering system, the letter “S” represents steel fibers, followed by a number indicating the volume fraction of steel fibers, set at 0%, 1%, and 2%, respectively.

Li et al. [27] (pp. 673–684) conducted high-temperature dynamic compression tests on SFRC specimens sharing the same dimensions, fiber volume content, and impact velocity as those in the current study. The raw materials used in their experiment included P.O. 42.5 ordinary Portland cement, pebbles, and crushed stones (particle size: 5–10 mm), medium-coarse river sand (fineness modulus: 2.8–3.0), polycarboxylate high-performance water reducer, and copper-plated steel fibers. The mix design for the SFRC is detailed in Table 3. The specimen designation C60 signifies plain concrete without steel fibers and a matrix strength of 60 MPa. C60S1 refers to SFRC with 1% fiber content, while C60S2 represents the specimens with 2% fiber content.

**Figure 2:**
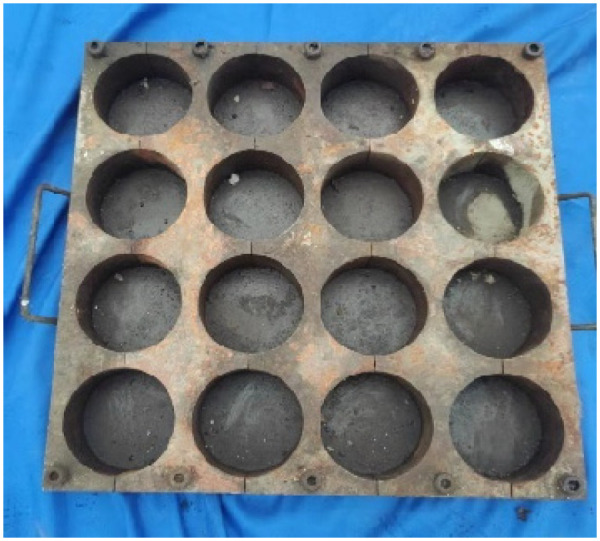
Specimen casting mold.

**Figure 3:**
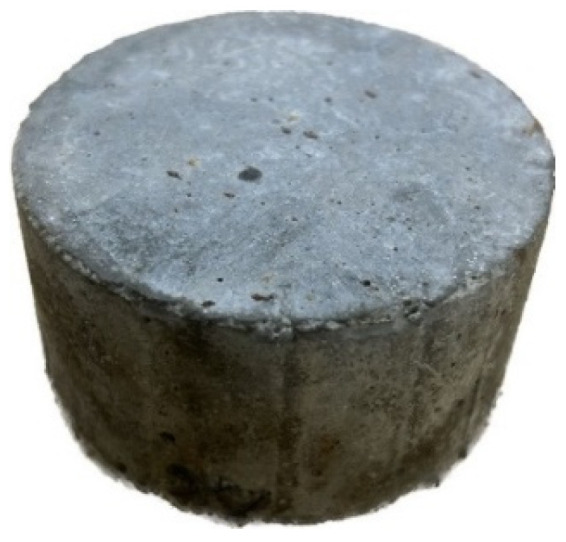
Cured specimen.

Based on the diameter of SHPB, the flat cylindrical specimens for SFRCC were set at 70 mm in diameter with a height of 35 mm. The specimen preparation process is outlined as follows: The process began with the accurate measurement of the cement, silica fume, water, water reducer, and steel fibers. Pour the measured cement and silica fume into a mixer and dry mix for three minutes to achieve a uniform blend of the dry components. Subsequently, mix the measured water and water reducer, then add them to the mixer in three batches. After each water addition, mix for 2 min to ensure the mortar consistency meets the required standards. Lastly, gradually add the steel fibers into the mixed mortar and stir for 5 min to ensure their uniform dispersion throughout the matrix [28]. A release agent was applied to the inner surfaces of the casting molds to facilitate the demolding process (Figure 2). After the fibers were uniformly distributed in the slurry, the mixture was poured into the molds and vibrated using a vibrating table to remove any air bubbles. Following the vibration, the surfaces of the specimens were leveled. After curing at room temperature for 24 h, the specimens were demolded and labeled. The specimens were then subjected to standard curing in a curing chamber (temperature: 20 ± 0.5 °C, relative humidity: 95 ± 5%) for 28 days. After the curing period, the specimens were polished to ensure that the flatness and parallelism of both end surfaces were within 0.02 mm. The cured specimens are depicted in Figure 3. To ensure the statistical significance of the test results, a minimum of five valid repeated specimens were prepared and successfully tested for each working condition in this study. The mechanical property data presented in the text represent the arithmetic mean of the valid results obtained from each group.

The performance of the SFRCC specimens in this study is compared with that of the SFRC specimens reported in reference [27]. To ensure the validity of this comparative analysis, the two types of specimens were matched in key parameters, including identical geometric dimensions, the same volume fraction of steel fibers, and the same SHPB impact velocity. It should be noted, however, that due to fundamental differences in the matrices of the two materials, inherent variations exist in their mix proportions, aggregate types, and particle sizes. These differences arise from the intrinsic characteristics of each material system. The purpose of this study is to examine the differences in high-temperature dynamic performance between the two materials under conditions where the fiber content is identical but the matrices differ. Therefore, the comparison results provide insights into the influence of matrix characteristics on overall performance.

### 2.2. Heating Scheme

**Figure 4:**
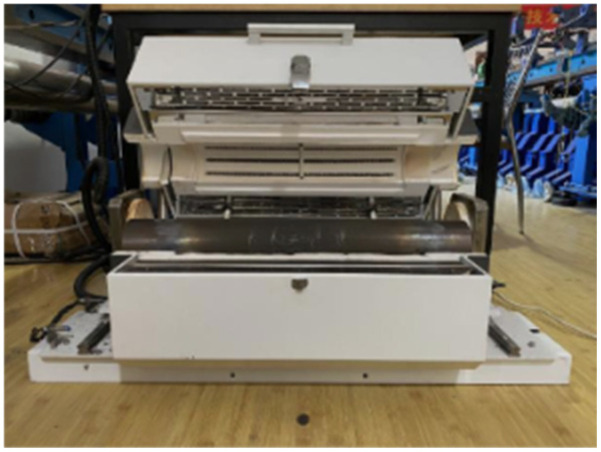
Resistance-type high-temperature heating furnace.

In this experiment, a resistance-type high-temperature furnace (Jiangshui Dynamic Mechanics Laboratory, Hefei, China) (Figure 4) was employed to heat the specimens. The furnace features a central explosion-proof chamber specially designed to accommodate the specimens, constructed with a 10 mm thick, heat-resistant stainless steel tube. This design effectively isolates any fragments that may be generated by the specimens at high temperatures, thereby safeguarding the furnace body from damage. The equipment is capable of withstanding high-temperature environments up to 1000 °C.

**Figure 5:**
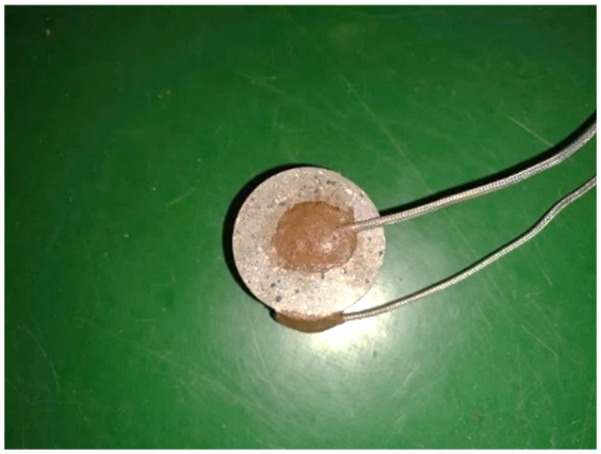
Layout of temperature measuring thermocouples.

During the testing process, it is crucial to ensure that the entire specimen reaches the target temperature uniformly, thereby eliminating any internal temperature gradients. The core principle in designing the heating protocol is to ensure uniform internal temperature within the specimen, with the required duration primarily governed by thermal-physical parameters such as the thermal conductivity and specimen size [29]. The thermal conductivity of typical cement-based materials is approximately 1.4 W/(m·K), whereas the incorporation of steel fibers slightly increases it to around 1.8 W/(m·K). According to heat conduction theory, higher thermal conductivity facilitates faster internal heat transfer, thereby reducing the time required to reach thermal equilibrium. As a result, the theoretical holding time for SFRCC specimens should be shorter than that for ordinary concrete. The selection of the plain matrix specimen (S0) for testing aims to determine the heating parameters corresponding to the longest required duration. Since the addition of fibers generally enhances thermal conductivity, a time scheme validated using the matrix alone ensures adequate temperature uniformity, even for specimens with slower thermal response. A 2 mm diameter hole, 17.5 mm deep, was carefully drilled at the center of the top surface of the specimen. A thermocouple was inserted into the hole and sealed with fire-resistant clay to precisely measure the temperature at the center of the cylinder. Additionally, another thermocouple was affixed to the side surface of the specimen to monitor the surface temperature. The positioning of the thermocouples for temperature assessment is illustrated in Figure 5. For the center-symmetric cylindrical specimens, once the temperature at the specimen’s center reaches the designated target temperature for the experiment, it is assumed that the entire specimen has uniformly attained that temperature.

In this study, after 28 days of standard curing, the internal temperature of the specimens was measured by inserting thermocouples into pre-drilled holes rather than embedding them during casting. This approach was adopted for two primary reasons. First, it ensures the standardization and consistency of specimen preparation. High-frequency vibration during specimen forming may damage thermocouple junctions or alter the local slurry density and the uniform distribution of steel fibers, introducing uncontrollable variables and compromising the accuracy of mechanical property tests. Second, this method enhances the accuracy and reliability of temperature measurements. Pre-embedded thermocouples may experience moisture ingress or corrosion at the junctions and wires during long-term curing, leading to signal drift or failure during high-temperature testing. Installing thermocouples immediately before the temperature-rise test ensures optimal sensor performance and, thus, more reliable temperature data.

The drilling and subsequent sealing with refractory mortar may introduce minor local disturbances to the thermal field and mechanical properties. However, their influence was minimized through careful experimental design. The borehole diameter was only 2 mm, with a depth equal to half of the specimen height. For a centrally symmetric cylindrical specimen, cavities of such small dimensions have a negligible effect on the overall heat conduction path. Moreover, the high-temperature refractory mortar used exhibits excellent thermal stability, and its thermal conductivity is comparable to that of cement-based materials, thereby effectively reducing heat-flow distortion around the boreholes. Thermocouple readings at both the specimen center and surface were cross-checked to confirm a uniform internal temperature during the holding stage. Consequently, when this validated heating scheme is applied to all intact and undamaged formal specimens, the resulting thermal exposure conditions are reliable and consistent.

**Figure 6:**
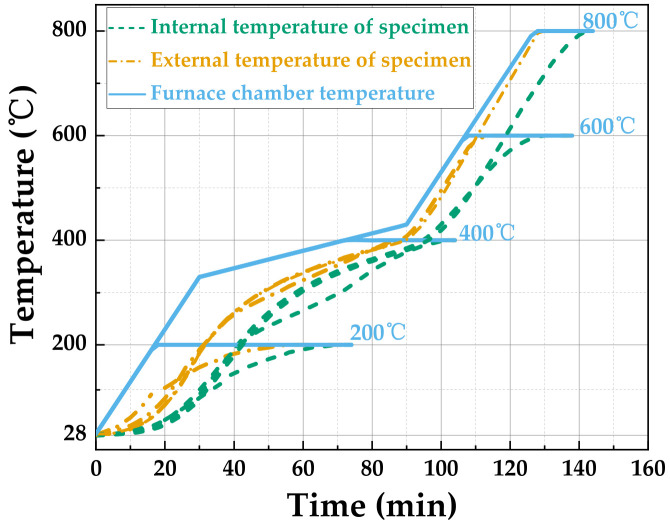
Heating schemes.

Following the completion of the specimen’s insulation process, both sides of the furnace doors were opened, and the incident and transmissive rods were introduced and aligned with the specimen. It is important to note that some heat loss may occur during this procedure. To mitigate this impact, the upper temperature limit of the furnace chamber for each target temperature was set 10 °C higher than the intended temperature. In particular, a lower heating rate is employed between 350 °C and 400 °C to prevent cracking of cement-based materials caused by rapid water evaporation and the loss of crystalline water in this temperature range, thereby ensuring the integrity of the specimen [30]. The holding time at each temperature is determined such that the thermocouple reading at the center of the specimen reaches and stabilizes within ±5 °C of the target value, achieving a balance between experimental efficiency and temperature uniformity. Based on these principles, the final heating protocols for 200 °C, 400 °C, 600 °C, and 800 °C, including the heating rate and holding time at each target temperature as determined through experimental testing [31,32], are summarized in Figure 6 and used as the unified standard for all high-temperature tests in this study.

### 2.3. Experimental Device for Dynamic Compression

**Figure 7:**
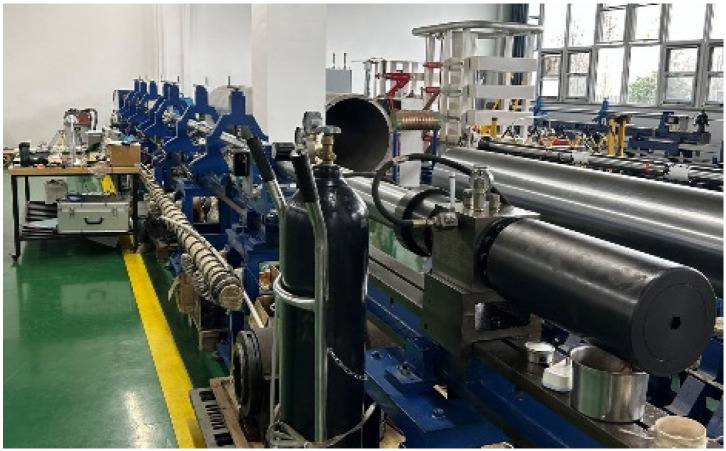
SHPB experimental equipment.

**Figure 8:**
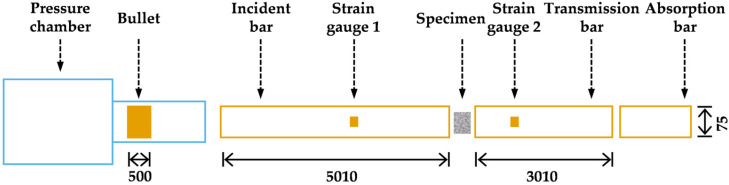
SHPB diagram (unit: mm).

SHPB with a 75 mm diameter (Jiangshui Dynamic Mechanics Laboratory, Hefei, China) (Figure 7) was utilized to perform high-temperature dynamic compression tests on SFRCC. This system primarily comprises a bullet (500 mm in length), an incident bar (5013 mm), a transmission bar (3013 mm), an absorber bar, a velocity measurement device, and a data processing system. The bullet, incident bar, and transmission bar are made of 60Si2Mn spring steel, each possessing an elastic modulus *E* of 210 GPa, a density *ρ* of 7800 kg/m^3^, and a wave velocity *C* of 5170 m/s, thereby maintaining consistent wave impedance *ρC*. Throughout the experiment, the bars are maintained in an elastic state. In the dynamic compression test, compressed gas propels the bullet with an initial velocity *v*_0_ and length *L*_0_ to impact the incident bar, creating an impact load. This action generates an incident pulse ***p****_I_*(*t*) within the incident bar. The specimen deforms under the influence of the incident pulse, producing a reflected return pulse ***p****_R_*(*t*) back to the incident bar and transmitting a transmission pulse ***p****_T_*(*t*) to the transmission bar. The velocity of the bullet *v*_0_ is monitored by a velocity measurement system comprising a parallel condenser light source, a phototube, an amplifier circuit, and a time interval meter. Pulse signals are captured and recorded using strain gauges, ultra-dynamic strain meters, and transient waveform recorders attached to the bars. The overall schematic of the SHPB apparatus is presented in Figure 8.

**Figure 9:**
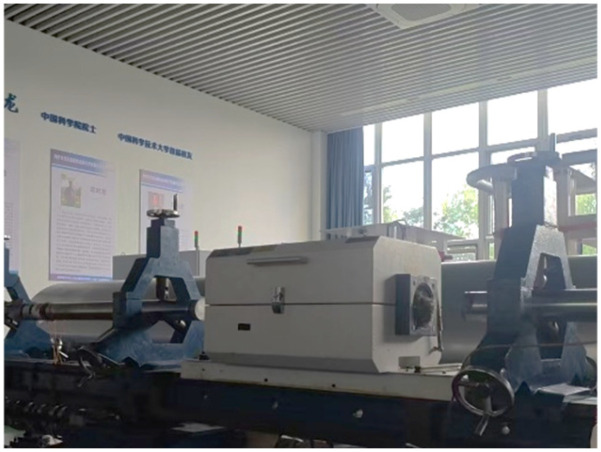
Combined SHPB and high-temperature heating furnace.

**Figure 10:**
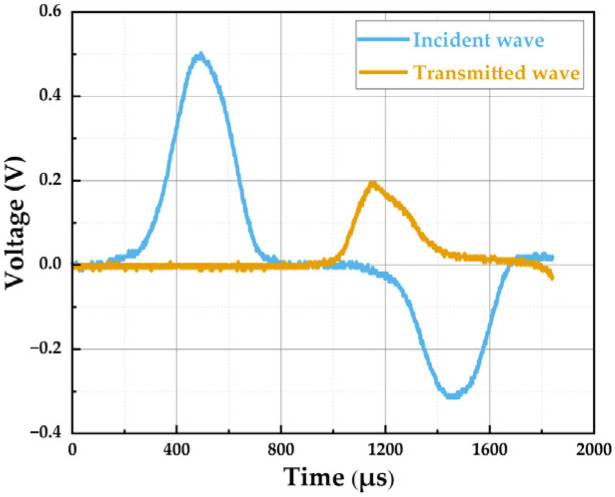
Original waveforms.

**Figure 11:**
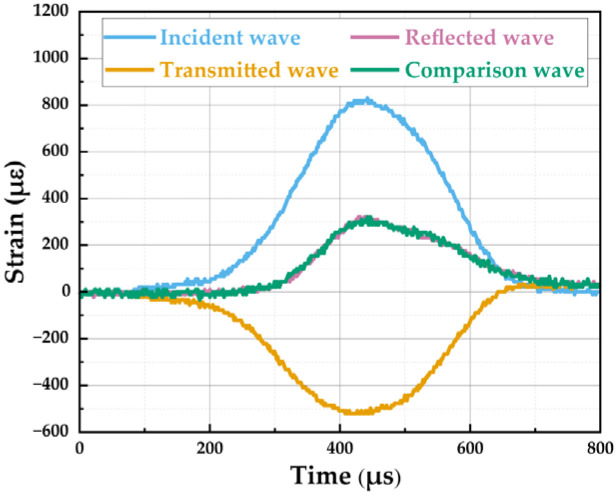
Aligned waveforms.

After integrating the SHPB system with the resistance-type high-temperature furnace (Figure 9), aligning the experimental equipment, affixing strain gauges, and configuring parameters for the dynamic test system, the dynamic compression test on SFRCC specimens under high-temperature conditions can commence. During the heating and holding phases of the specimen within the furnace, the closure of the two side doors helps minimize heat dissipation. Following the completion of the holding period, the drive unit is utilized to laterally open the two side doors, enabling swift insertion of the incident and transmission bars into the furnace. Concurrently, a pre-tension force is applied to ensure secure contact with the specimen. The high-pressure nitrogen gas in the cylinder is then released to propel the bullet, generating impact loading, enabling real-time dynamic compression loading at the designated target temperature. During the impact loading process, strain sensors affixed to the incident and transmission bars continuously capture the dynamic response signals (Figure 10). These signals are then processed by a signal processing system to segregate the characteristic waveforms into incident, reflected, and transmitted waves. According to the one-dimensional stress wave theory of the SHPB test, the dynamic response of the specimen can be determined by measuring the strain pulses in the incident and transmitted bars. A key prerequisite for this calculation is that the specimen reaches stress equilibrium during deformation, meaning the forces at both ends of the specimen must be equal. Following alignment of the waveforms in the time domain and determining a shared starting point for all waveforms (Figure 11), the transmitted wave curve is juxtaposed with the theoretical superimposed waveform, composed of incident and reflected waves, which is:

(1) $$p_{I}\left(t\right)+p_{R}\left(t\right)=p_{T}\left(t\right)$$

The approximate waveform alignment serves to confirm that the specimen adheres to the stress balance condition during the transmission of the impact load, thereby validating the test setup’s reliability and the efficacy of the acquired data. The stress waves were processed with the three-wave method to calculate the axial stress $\sigma_{s}$, strain rate ${\dot{\varepsilon }}_{s}$, and strain $\varepsilon_{s}$ of specimen [33] (pp. 5–10):

(2) $$\sigma_{s}=\frac{EA}{A_{s}}\left[p_{I}\left(t\right)+p_{R}\left(t\right)\right]=\frac{EA}{A_{s}}p_{T}\left(t\right)$$

(3) $${\dot{\varepsilon }}_{s}=\frac{2C_{0}}{l_{s}}\left[p_{I}\left(t\right)-p_{T}\left(t\right)\right]=-\frac{2C_{0}}{l_{s}}p_{R}\left(t\right)$$

(4) $$\varepsilon_{s}=\frac{2C_{0}}{l_{s}}\int_{0}^{t}\left[p_{I}\left(t\right)-p_{T}\left(t\right)\right]dt=-\frac{2C_{0}}{l_{s}}\int_{0}^{t}p_{R}\left(t\right)dt$$

where *A* represents the cross-sectional area of the bar, *A_s_* and *l_s_* denote the initial cross-sectional area and length of the specimen, respectively, and *t* is the time.

Upon completion of the impact loading, the incident and transmission bars are retracted, and the explosion-proof chamber is left to naturally cool in ambient air. Once the temperature within the chamber decreases to around 400 °C, it is removed from the furnace and further cooled using a fan. After the chamber reaches room temperature, any debris within is cleared, preparing the setup for subsequent impact tests.

## 3. Results

### 3.1. Test Results

Given the inherent data dispersion in high-strain-rate and high-temperature coupled tests, a minimum of five valid repeated tests were conducted for each condition under various temperatures, fiber contents, and loading rates to ensure result reliability. The data presented in this study reflect the arithmetic mean of valid measurements obtained for each group, with all coefficients of variation maintained within 10%, ensuring both repeatability and statistical significance. The stress–strain curve depicted in the figure corresponds to the most representative individual test result. This section initially presents an overall depiction of the dynamic compression performance of SFRCC and SFRC specimens under varying temperatures and loading rates. The test outcomes indicate that temperature predominantly influences material behavior. As temperature increases, the dynamic peak stress of all specimens notably decreases, while the peak strain generally rises. The inclusion of steel fibers effectively improves the mechanical properties of the materials, as demonstrated by the higher peak stress and toughness of fiber-reinforced specimens compared to their corresponding matrix specimens. Significantly, the performance of SFRCC and SFRC differs across temperature ranges: from 200 to 400 °C, SFRCC demonstrates superior strength and toughness, whereas from 600 to 800 °C, SFRC shows a relative advantage attributed to the stability provided by its aggregate system.

**Figure 12:**
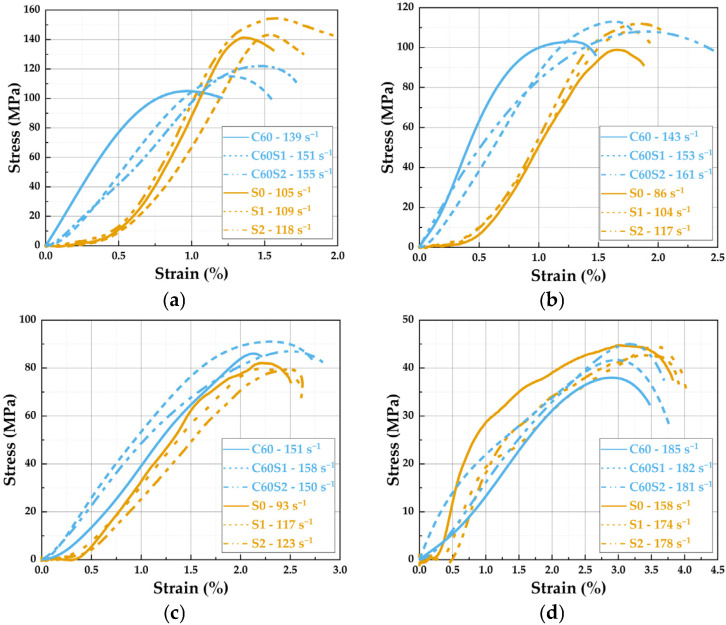
Comparison of dynamic compression stress–strain curves between SFRCC and SFRC: (**a**) 200 °C; (**b**) 400 °C; (**c**) 600 °C; (**d**) 800 °C.

The dynamic compression findings of SFRCC and SFRC specimens with identical steel fiber volume content under elevated temperatures are summarized in Table 4 and Table 5, for loading rates of 7 m/s and 8 m/s, respectively, and the comparison of stress–strain curves is illustrated in Figure 12. The mechanical properties of SFRCC material significantly improve with an increase in steel fiber content. At 200 °C, the peak stress of S2 is about 20% higher than that of S0. At 400 °C, S2 demonstrates outstanding ductility, with its peak strain approximately 30% higher than that of the matrix specimen. This suggests that the incorporation of steel fibers effectively mitigates the brittle failure of ECC materials under high-temperature conditions [34]. Between 600 °C and 800 °C, the stress of SFRCC notably diminishes, yet S1 and S2 specimens still retain some residual compressive strength. The stress–strain curves of these specimens exhibit a smoother trend, signifying that the fiber network effectively delays material failure. The influence of steel fiber content on the dynamic stress–strain responses of SFRC specimens is comparatively less pronounced when juxtaposed with the SFRCC series of specimens [35]. The peak stress increment in the C60S2 specimen is modest, standing only about 10% higher than that of the C60 specimen at 200 °C. However, the strain of the C60S2 specimen demonstrates a notable improvement, particularly evident at 400 °C, where it surpasses that of the C60 specimen by approximately 25% but remains lower than that of the S2 specimen. As the temperature escalates to 800 °C, the stress–strain curve of the C60S2 specimen sharply declines, indicating that the behavior at high temperatures of SFRC specimens lags behind that of SFRCC specimens.

In this experiment, across all target temperatures, the peak strain of SFRCC specimens exceeded that of the corresponding SFRC counterparts. Specifically, at 600 °C, the strain of the S2 specimen surpassed that of the C60S2 specimen by approximately 35%, underscoring the ductility advantages of SFRCC for design considerations [36]. While SFRCC demonstrated comparable or slightly lower dynamic peak stress compared to SFRC, its exceptional strain capacity translated into enhanced energy absorption performance. The notable disparity in high-temperature performance between the two materials was most pronounced around 400 °C, showcasing the predominant ductility of SFRCC. As the temperature elevated to 800 °C, both materials witnessed a significant decrease in strength. Nevertheless, the SFRCC specimens managed to preserve a certain level of strain capacity, whereas the plastic deformation capacity of the SFRC specimens was nearly depleted.

**Figure 13:**
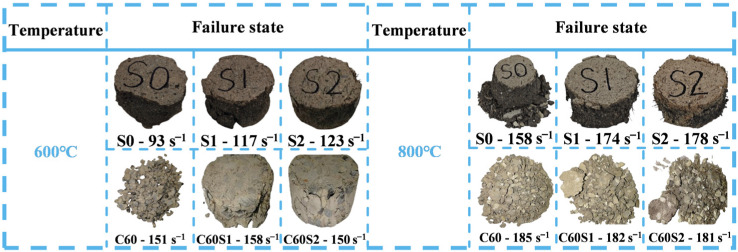
Comparison of failure states between SFRCC and SFRC specimens.

The comparison of failure states between SFRCC and SFRC specimens at elevated temperatures is depicted in Figure 13. The inertial effect influences the dynamic process of crack propagation, while strain-rate hardening significantly increases the yield stress of both the matrix and the fibers. Together, these mechanisms govern the mechanical behavior and failure mode of the material under impact loading [37,38]. At 600 °C, the S0 specimen maintained overall integrity but showed noticeable edge spalling. The occurrence of surface cracks decreased in the S1 and S2 specimens, implying the initiation of the fiber reinforcement effect. Particularly in the S2 specimen, enhanced overall integrity was evident with only localized spalling. With the temperature rising to 800 °C, the degree of fragmentation significantly increased in the S0 specimen, accompanied by clear block disintegration at the edges, signifying matrix degradation predominating the failure process [39]. The alteration in the failure mode of the S2 specimen was minimal as the steel fibers’ bridging effect effectively delayed complete failure in SFRCC. The C60 specimen experienced complete fragmentation at 600 °C, whereas the incorporation of steel fibers notably enhanced the overall integrity of the C60S1 and C60S2 specimens, resulting in surface cracks and side spalling and cracking. These specimens exhibited a more uniform crack distribution, indicative of steel fibers effectively hindering concrete concentration failure at elevated temperatures [8]. The progressive failure mode exhibited by SFRCC is quantitatively reflected in the shape of its stress–strain curve. As shown in Figure 12d, at 800 °C, the stress–strain curve of the S2 specimen displays a gradual, ductile decline after reaching the peak stress, indicating a sustained capacity for energy absorption during failure. In contrast, the curve for the C60S2 specimen shows a sharp, abrupt post-peak drop, typical of brittle failure. By comparing the slopes of the descending branches, the progressive nature of the failure can be described semi-quantitatively. The relatively gentle post-peak decline observed for SFRCC directly demonstrates its superior ductility and damage tolerance. In conclusion, SFRCC displayed a ductile failure behavior mode superiority at 600 to 800 °C, making it suitable for ductility design in high-temperature applications like fire-resistant structures. SFRC generally sustained stable performance at 600 °C but experienced rapid deterioration at 800 °C, emphasizing the necessity to minimize prolonged exposure to high temperatures in service. The qualitative analysis of the stress–strain curve and failure mode discussed above underscores the performance differences between SFRCC and SFRC under high-temperature dynamic loading. To further quantify and compare the dynamic mechanical responses of these materials, the following sections will present a systematic comparative analysis, focusing on key parameters such as strain rate sensitivity, dynamic peak stress, strain, and toughness. This analysis seeks to elucidate the underlying mechanisms responsible for these performance differences.

### 3.2. Comparative Analysis of Dynamic Compression Properties

#### 3.2.1. Strain Rate

**Figure 14:**
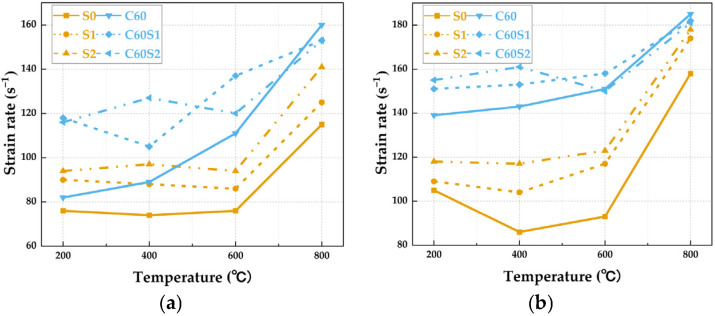
Variation in strain rate with temperature for SFRCC and SFRC specimens under different loading rates: (**a**) 7 m/s; (**b**) 8 m/s.

Strain rate is a key parameter that characterizes the dynamic response of materials. This section provides a comparative analysis of the strain-rate evolution of SFRCC and SFRC under different temperatures and loading rates to elucidate differences in their sensitivity and adaptability to impact loading. The strain rate trend with temperature for various SFRCC and SFRC specimens under different loading rates is visualized in Figure 14. Notably, the SFRCC materials in the series display pronounced strain rate sensitivity. At a loading rate of 7 m/s, the S0 specimen maintained a relatively low strain rate, exhibiting a gradual slope around 600 °C, indicating a limited sensitivity of the unmodified ECC matrix to temperature, despite modest improvements in dynamic performance [40]. In contrast, the S1 and S2 specimens showcased enhanced dynamic response capabilities. Particularly, the S2 specimen exhibited a strain rate of 140 s^−1^ at 600 °C, representing a 55.6% increase compared to S0. Notably, the strain rate elevation of the S2 specimen at high temperatures was notably lower than in the medium-temperature range, suggesting the steel fiber network’s efficacy in sustaining stress transfer even under extreme temperatures. Upon increasing the loading rate to 8 m/s, the S0 specimen displayed an anomalous decline in strain rate at 400 °C, likely attributed to the temporary closure of microcracks within the matrix. Conversely, the S2 specimen demonstrated a consistent linear growth trend, with the ratio of strain rate at 8 m/s to that at 7 m/s stabilizing between 1.07 and 1.15. This confirms that the fiber bridging effect continues to effectively sustain stress transfer under dynamic loading conditions [41,42].

The strain rate response of SFRC materials demonstrates a nonlinear behavior. The strain rate of the C60S1 and C60S2 continues to rise with increasing temperature, with the most notable escalation observed at 800 °C. This results from the combined influence of high-temperature-induced debonding at the fiber–matrix interface and the amplifying effect of dynamic loading. This phenomenon aligns with the interface degradation mechanism discussed in reference [43]. Upon elevating the loading rate to 8 m/s, the degradation process of the concrete material accelerated. Particularly, the C60S2 specimen attained an exceptionally high strain rate of 180 s^−1^ at 800 °C, with a ratio to the 7 m/s rate reaching as high as 1.2, markedly surpassing the S2 specimen. This highlights the heightened sensitivity of SFRC to the magnifying impact load effect. By comparing the dynamic constitutive response of the two materials, it can be observed that within the same temperature range, the strain rate increase in SFRC can reach up to 120%, significantly higher than SFRCC’s 66.7%. This difference mainly stems from the fundamental distinction between the concrete matrix and the cement matrix. Additionally, SFRCC exhibits a turning point at 600 °C, indicating a broader temperature stability range. The gradual curve characteristic of SFRCC within the 600 °C range reflects an energy-dissipating mechanism primarily dominated by fiber pull-out, while the oscillating curve of SFRC reflects a mixed failure mode of matrix fracturing and fiber rupture [44]. The above analysis of the strain-rate response indicates that SFRCC and SFRC exhibit distinctly different deformation mechanisms under dynamic loading. These differences inevitably influence their dynamic load-resisting capacity, particularly the dynamic peak stress. The following section provides a detailed discussion of this relationship.

#### 3.2.2. Dynamic Peak Stress

**Figure 15:**
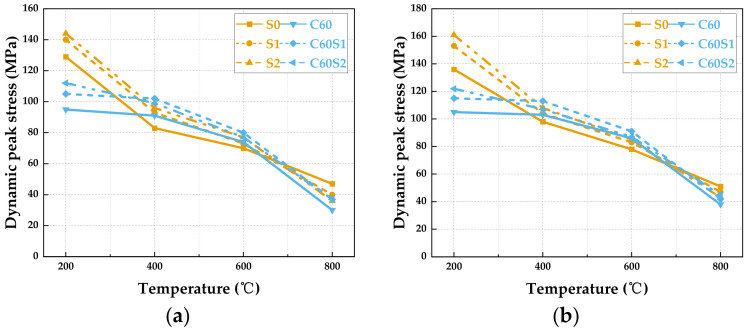
Variation in dynamic peak stress with temperature for SFRCC and SFRC specimens under different loading rates: (**a**) 7 m/s; (**b**) 8 m/s.

Dynamic peak stress directly reflects the ability of material to resist dynamic compressive loading. The following analysis examines the effects of temperature and fiber content on the dynamic strength degradation of the two materials, comparing their performance advantages and limitations across different temperature ranges. Figure 15 shows the fluctuation of dynamic peak stress with temperature for SFRCC and SFRC specimens under different loading rates. At a loading rate of 7 m/s, the SFRCC series materials exhibit notable mechanical performance gradients. The S0 specimen reaches a dynamic peak stress of 136 MPa at 200 °C, markedly surpassing the SFRC specimens. The qualitative conclusion that SFRCC exhibits superior high-temperature stability is quantitatively supported by its residual strength ratio. As shown in Table 4, the dynamic peak stress of the S2 specimen of SFRCC at 800 °C is 43 MPa, corresponding to approximately 26.7% of its peak stress at 200 °C (161 MPa), indicating that it retains more than one quarter of its original strength. In comparison, the dynamic peak stress of the C60S2 specimen of SFRC at 800 °C is 42 MPa, which corresponds to 34.4% of its peak stress at 200 °C (122 MPa). Although the absolute strengths of the two materials at 800 °C are comparable, SFRCC undergoes a more gradual strength degradation from a substantially higher initial strength, and its failure mode remains progressive (Figure 13), whereas SFRC exhibits severe fracturing. This combined assessment of strength retention and failure behavior quantitatively demonstrates the superior ability of SFRCC to maintain structural integrity under extreme high-temperature conditions.

In contrast to SFRCC, SFRC materials exhibit a distinct degradation trend at a rate of 7 m/s. At 200 °C, the dynamic peak strength of SFRCC materials generally surpasses that of SFRC specimens. As the temperature escalates to 400 °C and 600 °C, SFRC displays more pronounced dynamic compressive strength. By 800 °C, the dynamic peak stress of the S0 specimen surpasses that of the other five specimen types, mainly due to the absence of bond failure between the coarse aggregate and the matrix. The compact matrix structure exhibits enhanced thermal stability and mechanical performance at elevated temperatures, enabling it to uphold robust resistance to dynamic loads in high-temperature environments [45] (pp. 751–768). In contrast, the impact strengths of the S1, S2, C60, C60S1, and C60S2 specimens show relatively similar levels at 800 °C. Despite the inherent experimental dispersion, the performance degradation trends of the SFRCC specimens at different temperatures remain consistent and repeatable. This systematic variation provides strong evidence that the temperature-dependent evolution of fiber–matrix interface properties, such as effective bridging at low and medium temperatures and interface debonding at high temperatures, is the key mechanism driving the observed strength degradation. The above analysis reveals the distinct temperature-dependent evolution of dynamic strength in SFRCC and SFRC. However, the dynamic mechanical performance of materials is reflected not only in their load-bearing capacity but also in their deformation capacity [46].

#### 3.2.3. Dynamic Peak Strain

**Figure 16:**
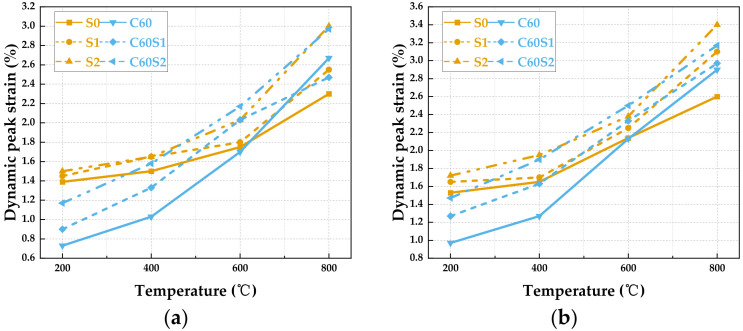
Variation in dynamic peak strain with temperature for SFRCC and SFRC specimens under different loading rates: (**a**) 7 m/s; (**b**) 8 m/s.

Dynamic peak strain characterizes the maximum deformation a material can withstand before failure and serves as a key indicator of its ductility. This section systematically analyzes the variation in the deformation capacity of SFRCC and SFRC with temperature and clarifies the ductility characteristics of both materials under elevated temperatures. The dynamic peak strain variation with temperature for SFRCC and SFRC specimens under various loading rates is depicted in Figure 16. At an impact velocity of 7 m/s, the SFRCC series materials exhibit typical temperature-dependent deformation characteristics. For instance, the S0 specimen demonstrates a dynamic peak strain of 1.39% at 200 °C, rising to 2.30% at 800 °C, marking a 165% increase. In contrast, the S1 and S2 specimens showcase enhanced deformation capabilities, with the S2 specimen achieving a peak strain of 3.00% at 800 °C, surpassing the S0 specimen by 30%. Furthermore, the strain growth rate of S1 and S2 between 400 °C and 600 °C surpasses that of S0 significantly, indicating that fiber modification notably augments the material’s plastic deformation abilities within the medium–high temperature range [47]. Conversely, SFRC materials display heightened temperature sensitivity under identical loading conditions. For instance, the initial strain of the C60 concrete matrix at 200 °C amounts to 0.73%, escalating to 2.67% at 800 °C, reflecting a 265% increment. The deformability of C60S1 and C60S2 is further pronounced, with C60S2 reaching a peak strain of 2.97% at 800 °C, exceeding the C60 specimen by 10%. Moreover, the strain growth rate of SFRC materials notably decelerates beyond 600 °C, suggesting that the materials are nearing their plastic deformation threshold.

In comparing the strain-temperature curves of the two materials, it is apparent that in the medium–high temperature range of 600 °C to 800 °C, the peak strain of SFRC materials generally surpasses that of the corresponding SFRCC specimens. Additionally, the reduction in the strain growth rate of SFRC materials from 600 °C to 800 °C is notably less than that of SFRCC, suggesting that the concrete matrix of SFRC materials exhibits prevailing deformation stability at elevated temperatures [48]. SFRCC relies on the progressive reconstruction damage mechanism of the SiO_4_ or AlO_4_ network structure to preserve its deformation capacity at high temperatures. As a result, the strain curve of the SFRCC specimens exhibits a smoother transitional trend under elevated temperatures. Based on the earlier analysis of dynamic peak stress and dynamic peak strain, it is evident that SFRCC and SFRC each demonstrate distinct advantages across different temperature ranges. SFRCC exhibits excellent strength and deformation capacity at low to medium temperatures. At high temperatures, however, SFRC maintains a certain level of strength due to the stability of its aggregate system, although its deformation capacity increases more gradually. To comprehensively evaluate the energy absorption efficiency of materials throughout the entire loading-to-failure process under dynamic conditions, it is necessary to introduce a composite index called toughness, which encompasses both strength and ductility.

#### 3.2.4. Toughness

**Figure 17:**
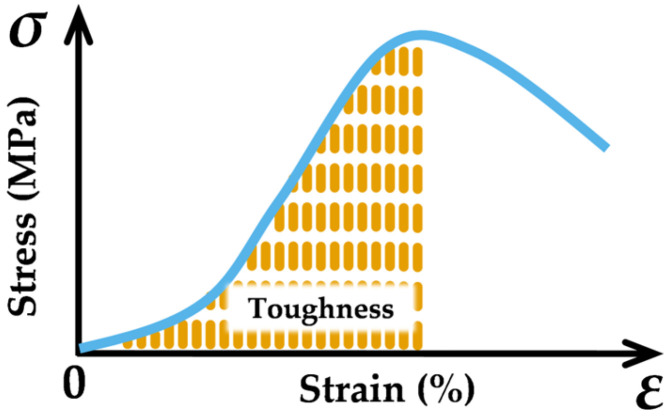
Definition of toughness.

Toughness, which reflects the capacity of material to absorb energy during fracture, is a combined measure of its strength and ductility. The toughness can be determined by integrating the area under the dynamic stress–strain curve from initial state to peak stress, as illustrated in Figure 17. A higher value of this indicator corresponds to dominant dynamic crack resistance in either SFRCC or SFRC.

**Figure 18:**
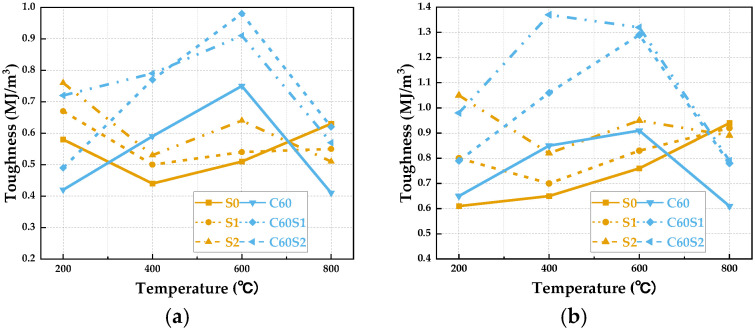
Variation in toughness with temperature for SFRCC and SFRC specimens under different loading rates: (**a**) 7 m/s; (**b**) 8 m/s.

The toughness of three types of SFRCC specimens first decreases and then increases as the temperature rises, whereas the SFRC specimens show an initial increase followed by a decrease, as depicted in Figure 18. Specifically, at 200 °C, the dynamic toughness of SFRCC specimens exceeds that of the SFRC specimens. At 400 °C and 600 °C, the dynamic stress peak toughness of the SFRC specimens exceeds that of the SFRCC specimens. For instance, considering the S1 and C60S1 specimens, under an 8 m/s loading rate at 200 °C, their dynamic stress peak toughness values are 0.79 MJ/m^3^ and 0.80 MJ/m^3^, respectively; at 600 °C, these values are 1.29 MJ/m^3^ and 0.83 MJ/m^3^. At 800 °C, the toughness of C60S1 and C60S2 specimens surpasses that of the S1 and S2 specimens, while the S0 specimens exhibit the highest dynamic stress peak toughness among the six specimen types.

The variation in toughness of SFRCC and SFRC with temperature primarily stems from the distinct thermal stability of their matrix microstructures, forming the core focus of this comparative study. In the temperature range of 400 °C at low to medium levels, the dense ECC matrix of SFRCC offers significant advantages. Its higher density, capability to control micro-cracking, and strong bonding at the fiber–matrix interface aid in effective stress transfer and crack delay [49]. Furthermore, the more uniform distribution of steel fibers in SFRCC compared to SFRC results in a more pronounced fiber bridging effect that enhances energy absorption capacity during dynamic loading. However, SFRC specimens exhibit relative fragility in this temperature range, prone to concentrated cracking under dynamic loads, leading to reduced toughness. Transitioning to the medium to high-temperature range of 400 °C to 600 °C brings about a transformation. Dehydration of hydration products and the loose microstructure of SFRCC cause disintegration at the matrix–fiber interface, diminishing the fiber reinforcement effect. Conversely, the concrete matrix of SFRC shows better stability at high temperatures, attributed to the presence of aggregates [50]. The minimal differences in thermal expansion coefficients between aggregates and cement paste help mitigate interface damage caused by thermal mismatch deformation. At extreme conditions up to 800 °C, the failure behavior of S0 specimens is chiefly characterized by uniform matrix softening without stress concentration from fibers. The plastic deformation and viscous flow processes in the matrix at elevated temperatures enhance energy absorption, demonstrating peak toughness before dynamic stress peaks.

In conclusion, despite inherent data dispersion within each group, the performance trends of SFRCC and SFRC at different temperatures are clear, consistent, and replicable. These distinct systematic patterns, such as the progressive failure of SFRCC at elevated temperatures and the notable performance decline of SFRC at extremely high temperatures, effectively confirm the earlier analysis regarding the fundamental mechanisms associated with matrix properties, fiber–matrix interface behavior, and aggregate effects.

## 4. Conclusions

This study performed dynamic compression tests on SFRCC under high-temperature conditions and compared the results with those of SFRC. The differences between the two materials were analyzed based on key parameters, including stress–strain curves, failure modes, dynamic peak stress, strain, toughness, and others, at various temperatures. The key conclusions are:

1. Under the same steel fiber content, SFRCC demonstrates enhanced overall performance under high-temperature conditions. The fiber–matrix synergy in SFRCC enables the material to maintain a gradual failure mode at 800 °C, retaining approximately 40% of its residual strength. In contrast, SFRC experiences a rapid decline in performance after 600 °C, resulting in a significant increase in its degree of fragmentation.
2. Under high-temperature conditions, as the loading rate increased from 7 m/s to 8 m/s, the S2 specimens displayed a stable strain rate growth trend and, after 600 °C, maintained a damage mode predominantly characterized by fiber pull-out. In contrast, SFRC materials experienced rapid degradation of the matrix–fiber interface at elevated temperatures, resulting in a larger increase in strain rate, accompanied by performance fluctuations.
3. The dynamic peak stress and toughness of SFRCC are considerably higher than those of SFRC within the low-temperature range of 200 °C to 400 °C. As the temperature increases to the medium–high range of 600 °C to 800 °C, SFRC exhibits excellent performance in terms of dynamic peak stress and toughness, due to the thermal stability advantage of its aggregate–matrix system.

The results of this study offer direct guidance for the design and evaluation of structures exposed to high temperatures or fire hazards, including industrial buildings, traffic tunnels, underground spaces, and high-rise buildings. For structural components requiring excellent ductility, crack resistance, and explosion resistance, SFRCC should be prioritized in environments where medium to low temperatures are expected. Its superior deformation capacity and progressive failure mode help prevent catastrophic brittle failure. For components that emphasize overall stability and residual strength at high temperatures, SFRC shows strong application potential in the 600–800 °C range. The comparative analysis presented in this study provides a performance-based framework for material selection in engineering practice. Designers can make informed trade-offs and optimization decisions between SFRCC and SFRC by considering factors such as fire risk levels, structural importance, and the specific performance requirements of a given project.

Although this study systematically reveals the performance differences between the two material types under high-temperature dynamic compression, it has certain limitations. The focus is primarily on uniaxial dynamic compression behavior, and future research could expand to include more complex stress states, such as dynamic tension and multi-axial loading, to better address the needs of comprehensive structural analysis. Additionally, this paper centers on the instantaneous mechanical response of materials at high temperatures, while long-term high-temperature creep behavior and specimen size effects remain important areas for further investigation. Furthermore, future work could incorporate advanced microscopic techniques, such as scanning electron microscopy (SEM) and X-ray tomography, to quantitatively examine the evolution of the fiber–matrix interface before and after exposure to high temperatures, thus providing deeper insights into the microscopic mechanisms that drive macroscopic performance differences.

## Figures and Tables

**Table 1:** Basic properties of steel fiber.

Length (mm)	Diameter (mm)	Aspect Ratio	Density (kg/m^3^)	Elastic Modulus (GPa)	Tensile Strength (MPa)
12	0.2	60	7800	210	3000

**Table 2:** Mass ratios of SFRCC specimens.

Specimen Type	Cement	Silica Fume	Water	Sand	Superplasticizer	Steel Fiber
S0	1	0.11	0.37	1.8	0.008	0
S1	1	0.11	0.37	1.8	0.008	1.0
S2	1	0.11	0.37	1.8	0.008	2.0

**Table 3:** Mixture ratios of SFRC specimens (kg/m^3^) [27].

Specimen Type	Cement	Sand	Stone	Water	Superplasticizer	Steel Fiber
C60	600	668	1002	180	9.0	0
C60S1	600	668	1002	180	9.0	78
C60S2	600	668	1002	180	9.0	176

**Table 4:** Results of dynamic compression experiments of SFRCC.

Number	Temperature	Loading Rate	Strain Rate	Dynamic Peak Stress	Dynamic Peak Strain	Toughness
	(°C)	(m/s)	(s^−1^)	(MPa)	(%)	(MJ/m^3^)
S0	200	7	76	129	1.39	0.58
		8	105	136	1.53	0.61
	400	7	74	83	1.50	0.44
		8	86	98	1.65	0.65
	600	7	76	70	1.75	0.51
		8	93	78	2.14	0.76
	800	7	115	47	2.30	0.63
		8	158	51	2.60	0.94
S1	200	7	90	140	1.45	0.67
		8	109	153	1.65	0.80
	400	7	88	93	1.65	0.50
		8	104	104	1.70	0.70
	600	7	86	73	1.80	0.54
		8	117	83	2.25	0.83
	800	7	125	40	2.55	0.55
		8	174	47	3.10	0.92
S2	200	7	94	144	1.50	0.76
		8	118	161	1.72	1.05
	400	7	97	96	1.65	0.53
		8	117	108	1.95	0.82
	600	7	94	77	2.03	0.64
		8	123	85	2.38	0.95
	800	7	141	36	3.00	0.51
		8	178	43	3.40	0.89

**Table 5:** Results of dynamic compression experiments of SFRC [27].

Number	Temperature	Loading Rate	Strain Rate	Dynamic Peak Stress	Dynamic Peak Strain	Toughness
	(°C)	(m/s)	(s^−1^)	(MPa)	(%)	(MJ/m^3^)
C60	200	7	82	95	0.73	0.42
		8	139	105	0.97	0.65
	400	7	89	91	1.03	0.59
		8	143	103	1.27	0.85
	600	7	111	74	1.70	0.75
		8	151	86	2.13	0.91
	800	7	160	30	2.67	0.41
		8	185	38	2.90	0.61
C60S1	200	7	118	105	0.90	0.49
		8	151	115	1.27	0.79
	400	7	105	102	1.33	0.77
		8	153	113	1.63	1.06
	600	7	137	80	2.03	0.98
		8	158	91	2.33	1.29
	800	7	153	37	2.47	0.62
		8	182	42	2.97	0.78
C60S2	200	7	116	112	1.17	0.72
		8	155	122	1.47	0.98
	400	7	127	99	1.58	0.79
		8	161	107	1.90	1.37
	600	7	120	77	2.17	0.91
		8	150	87	2.50	1.32
	800	7	153	37	2.97	0.57
		8	181	45	3.17	0.79

## Data Availability

The original contributions presented in this study are included in the article. Further inquiries can be directed to the corresponding author.

## Abbreviations

The following abbreviations are used in this manuscript:

SFRCC	Steel fiber-reinforced cementitious composites
SHPB	Split Hopkinson pressure bar
SFRC	Steel fiber-reinforced concrete
ECC	Engineered cementitious composites
